# Effect of Cooling Temperature on Crystalline Behavior of Polyphenylene Sulfide/Glass Fiber Composites

**DOI:** 10.3390/polym15153179

**Published:** 2023-07-26

**Authors:** Seo-Hwa Hong, Beom-Gon Cho

**Affiliations:** Chemical Materials R&D Department, Chassis & Materials Research Laboratory, Korea Automotive Technology Institute, 303 Pungse-ro, Pungse-myeon, Dongnam-gu, Cheonan-si 31214, Republic of Korea; shhong1@katech.re.kr

**Keywords:** poly (phenylene sulfide) (PPS), glass fiber, composites, injection molding, cooling temperature, crystallinity

## Abstract

Poly (phenylene sulfide) (PPS) is a super engineering plastic that has not only excellent rigidity and high chemical resistance but also excellent electrical insulation properties; therefore, it can be applied as an electronic cover or an overheating prevention component. This plastic has been extensively applied in the manufacture of capacitor housing as, in addition to being a functional and lightweight material, it has a safety feature that can block the electrical connection between the electrolyte inside and outside the capacitor. Moreover, the fabrication of PPS composites with high glass fiber (GF) content facilitates the development of lightweight and excellent future materials, which widens the scope of the application of this polymer. However, the crystallinity and mechanical properties of PPS/GF composites have been found to vary depending on the cooling temperature. Although extensive studies have been conducted on the influence of cooling temperature on the crystalline behavior of PPS-based composites, there has been limited research focused particularly on PPS/GF composites for capacitor housing applications. In this study, to apply PPS/GF composites as film capacitor housings, specimens were prepared via injection molding at different cooling temperatures to investigate the composites’ tensile, flexural, and impact energy absorption properties resulting in increases in mechanical properties at high cooling mold temperature. Fracture surface analysis was also performed on the fractured specimens after the impact test to confirm the orientation of the GF and the shape of the micropores. Finally, the crystallinity of the composites increased with higher cooling temperatures due to the extended crystallization time.

## 1. Introduction

Polyphenylene sulfide (PPS) is a high-performance thermoplastic polymer with excellent mechanical, thermal, and chemical properties, making it suitable for a wide range of industrial applications [1,2]. It exhibits excellent dimensional stability, flame resistance, and resistance to chemicals, solvents, and high temperatures. These properties make it a favored choice in various sectors, including the automotive, aerospace, electrical, and electronics industries [3,4].

PPS-based composites reinforced with glass fibers (GF) have garnered significant attention in recent years owing to their enhanced mechanical strength and dimensional stability [5,6,7]. These composites combine the desirable properties of PPS with the added reinforcement provided by GF, resulting in an improved performance [6,8]. Notably, PPS/GF composites have been extensively applied in the manufacturing of capacitor housings.

The crystalline structure of the PPS/GF composites plays a pivotal role in determining their properties [7,9]. The arrangement and morphology of the crystalline phase significantly affect various characteristics, including mechanical strength, electrical properties, and thermal stability [1,10,11]. Consequently, understanding the crystalline behavior of PPS/GF composites is essential to optimizing their processing conditions and tailoring their properties to satisfy specific application requirements.

Although the influence of cooling temperature on the crystalline behavior of PPS-based composites has been explored in previous studies, limited research has focused specifically on PPS/GF composites for capacitor housing applications [12,13,14,15,16].

Preliminary research on PPS/GF composites demonstrated the significance of their crystalline behavior concerning their properties. Jeong et al., investigated the effect of cooling temperature on the crystallinity and mechanical properties of PPS/GF composites and found that variations in cooling temperature led to changes in the degree of crystallinity and, consequently, affected the mechanical strength of the composites [17]. Similarly, Zuo et al., examined the influence of cooling rate on the crystalline structure and physical properties of PPS/GF composites, highlighting the importance of crystalline behavior in determining the physicochemical properties of the materials [18].

However, despite these valuable contributions, a comprehensive understanding of the crystalline behavior of PPS/GF composites in capacitor housing applications is still lacking. Thus, further investigation into the effect of the cooling temperature on the crystalline behavior of these composites is warranted to fill this research gap. Such knowledge is essential for optimizing the processing conditions and tailoring the properties of PPS/GF composites to meet the specific requirements of capacitor housing.

This study aims to bridge this gap by investigating the crystalline behavior of PPS/GF composites for capacitor housing applications, specifically focusing on the influence of cooling temperature. X-ray diffraction (XRD) and differential scanning calorimetry (DSC) were used to analyze the crystalline structure of the composites, including the degree of crystallinity, crystalline phase content, and crystal size. Additionally, the mechanical properties of the composites, including the tensile strength, flexural strength, impact absorbed energy, dynamic mechanical properties, and fiber alignment in the matrix, were evaluated to establish correlations with the observed crystalline behavior.

The findings of this study, coupled with preliminary research, contribute to a comprehensive understanding of the processing–structure–property relationships of PPS/GF composites for capacitor housing applications. This knowledge will facilitate the development of high-performance composites tailored to satisfy the stringent requirements of the electronics industry.

## 2. Materials and Methods

### 2.1. Preparation of PPS/GF Composites

**Figure 1:**
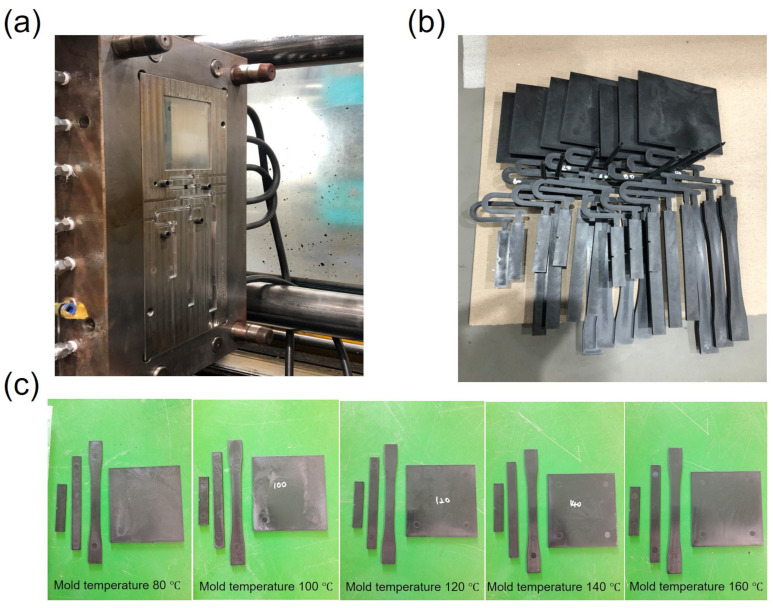
Photographs of (**a**) the steel mold for manufacturing of specimens to evaluate mechanical properties via injection molding process, (**b**) the resultant specimens after injection molding, and the PPS/GF 55 wt.% composites with different cooling temperatures are separated as shown in (**c**).

The PPS compounds reinforced with a 55% weight fraction of short glass fibers, which is currently being applied in electric vehicle (EV) capacitor housings, were supplied by Toray Advanced Composites Corporation (Tokyo, Japan). The plate samples were manufactured with the barrel set at 300 °C by injection molding as shown in Figure 1. By varying the injection mold temperature, which can be called the cooling temperature, an independent variable, the changes in the specific properties of the specimens according to the mold temperature were analyzed for the PPS/GF 55 wt.% composites. Importantly, the mold temperatures were set at 80, 100, 120, 140, and 160 °C, indicating that the injected polymer resin was cooled at these respective temperatures. Furthermore, insulation plates and a temperature control system were installed to stabilize the mold temperature. The molded product is shown in Figure 1a,b. Dog-bond-shaped and rectangular plate specimens were prepared for tensile, flexural, Izod, and drop-weight impact tests.

### 2.2. Characterization

#### 2.2.1. Morphology Analysis

The cross-sectional morphologies and orientation of GF in the composites were investigated using field-emission scanning electron microscopy (FE-SEM) (Helios 5 Hydra, Thermo Fisher Scientific, Waltham, MA, USA) at an accelerating voltage of 20 kV. The fractured tips were cut from the drop-weight impact-tested specimen for the fracture surface analysis. A cross-sectional surface analysis was performed by fracturing the samples after ultrasonic cleaning and coating them with Pt.

#### 2.2.2. Crystallographic Analysis

The crystallization behavior of PPS in the composites was analyzed via X-ray diffraction (XRD). XRD patterns of the PPS/GF composites were obtained in the 2θ range 5–50° at a rate of 5°/min, using the high-resolution X-ray diffractometer (MiniFlex600, Rigaku, Tokyo, Japan) with the Cu Kα target at 40 kV and 20 mA. The crystallite size of the composites at different reflection planes (110, 200) was calculated using Scherrer’s equation as follows:

(1) $$H=\frac{0.9\lambda }{\beta cos\theta }$$

where H is the height of the stacking layers, λ is the wavelength of the X-rays, and *β* is the full width at half maximum (2θ). Furthermore, the crystallinity index (CI) of the films was calculated using the following equation:

(2) $$\mathrm{CI}=\frac{A_{c}}{A_{c}+A_{a}}$$

where *A_c_* is the integrated area of the crystalline peaks, and *A_a_* is the integrated area under the amorphous halo. Thermal studies were conducted by differential scanning calorimetry (DSC) (DSC400, Perkin Elmer, Waltham, MA, USA). Nitrogen gas was purged into the chamber at a 20 mL/min flow rate during the DSC measurements. The crystallization isotherms of the samples were investigated by completing a heating–cooling cycle, where the samples were first heated from 30 °C to 350 °C at a ramping rate of 10 °C/min and then cooled to 30 °C at a ramping rate of 10 °C/min. The third scan was performed from 30 °C to 350 °C at a rate of 10 °C/min, during which the melting endotherms were identified. The degree of crystallinity (*Χ_c_*) was calculated as follows:

(3) $$X_{c}=\frac{\Delta H_{m}}{\Delta {H^{0}}_{m}}\times 100\%$$

where Δ*H_m_* and Δ*H^0^_m_* (150.4 J/g) are the enthalpies of the pure PPS matrix [15,19].

#### 2.2.3. Thermal Analysis

The thermal stabilities of the PPS/GF composites at different cooling temperatures were investigated using thermogravimetric analysis (TGA) (TGA4000, Perkin Elmer, Waltham, MA, USA) in a nitrogen environment at a flow rate of 20 mL/min. The samples were heat-treated from 30 °C to 850 °C at a ramp rate of 10 °C/min.

#### 2.2.4. Mechanical Testing

The tensile properties of the composites were measured using a tensile test machine (MINOS-300, MTDI, Daejeon, Republic of Korea) at a crosshead speed of 5 mm/min in accordance with the ASTM D638 standard [20]. The tensile strengths, strains, and elastic moduli of the specimens were evaluated seven times. Flexural tests of the composites were conducted based on the three-point bending mode according to ASTM D790 standards [21] (80 × 10 × 4 mm^3^ with a support span length of 52 mm). A constant crosshead speed of 2.8 mm/min was used for the bending tests of all samples. Flexural strength was calculated using the following equation:

(4) $$\mathrm{Fs}=\frac{3\mathrm{PL}}{2bd^{2}}$$

where Fs is the flexural strength, P is the maximum load, L is the length of the support span; b is the width, and d is the specimen thickness. Furthermore, the hardness of the composites was investigated using Rockwell and Shore D tests in accordance with the ASTM D785 and D2240 standards (100 × 100 × 3 mm^3^), respectively. The dynamic mechanical behavior of the composites was analyzed using dynamic mechanical analysis equipment (DMA8000, Perkin Elmer, Waltham, MA, USA) in accordance with the ASTM D4065 standard. The measurements were carried out in multi-frequency strain mode using a 3-point bending clamp within the temperature range from 30 °C to 200 °C, with a heating rate of 3 °C/min at 1 Hz. Finally, a drop-weight impact test was conducted to investigate the absorbed energy of the PPS/GF composites using a drop tower high-velocity impact tester (Instron, CEAST 9450, Norwood, MA, USA) with an impact speed of 2.72 m/s and an impact energy of 20 J in accordance with ASTM D7136 standards [22] (100 × 100 × 3 mm^3^).

## 3. Results

### 3.1. Crystallization Behavior of PPS/GF Composites

**Figure 2:**
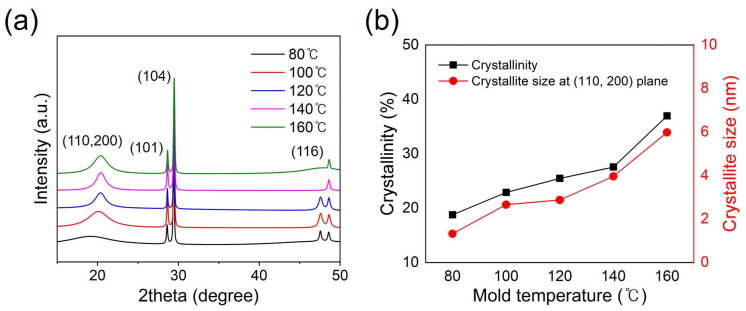
(**a**) XRD patterns of PPS/GF composites showing characteristic peaks at around 20° (110, 200 plane) corresponding to PPS peak; (**b**) shows crystallinity and crystallite size corresponding to the (110, 200) reflection plane of the PPS/GF composites with different cooling temperatures from XRD patterns.

As shown in Figure 2, the representative peak at ~20° is ascribed to the (110) and (200) crystalline planes of PPS [23,24]. As the cooling temperature increases from 80 °C to 160 °C, the peak intensity increases, showing a sharper peak than that at 80 °C; there is no crystalline peak at 20°, meaning that crystallization of PPS has not yet progressed at 80 °C. In other words, because the cooling temperature was relatively low, there was insufficient time for PPS crystallization to proceed. Therefore, as the cooling temperature gradually increases, the PPS peak intensifies and becomes most active at 160 °C; hence, there is sufficient time for the PPS chain to crystallize at a cooling temperature of 160 °C. Accordingly, the crystallinity (CI) and crystallite size of the PPS/GF composites increases by 100% and 353%, gradually as cooling the temperature increases from 80 °C to 160 °C, as listed in Table 1. In other words, the higher the cooling temperature, the more time it takes for the PPS chain to crystallize, which can result in an increase in crystallinity. In addition, an increase in the cooling temperature was confirmed to affect the crystallization of PPS through the behavior of the crystal size. Meanwhile, the two strong peaks at 28° and 30°, which are related to quartz (101) crystalline plane and hexagonal calcite (104) crystalline planes of calcium carbonate, respectively, can be seen in all cases as having a stable initial shape [25,26,27]. In addition, the 48° peak is related to the (116) calcite plane. Calcium carbonate is widely used as a filler for surface coatings, leading to hydrophobicity or mechanical and thermal properties, particularly non-flammability, in polymer composites.

### 3.2. Thermal Properties of PPS/GF Composites

**Figure 3:**
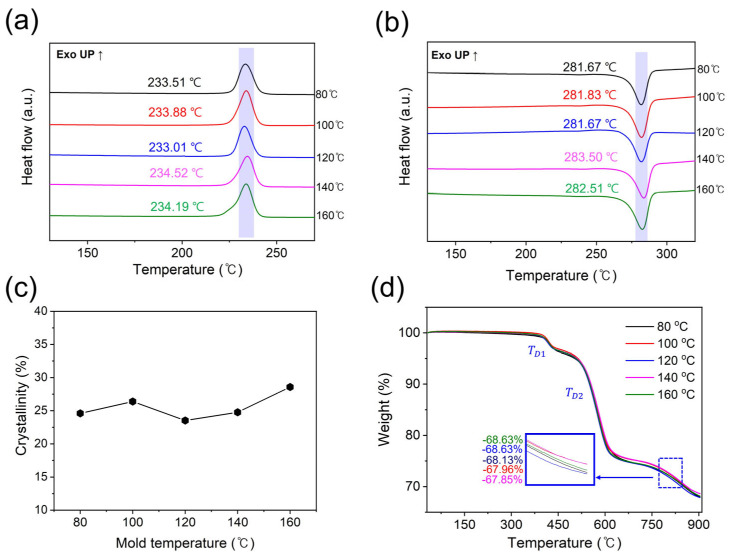
Non-isothermal DSC scans of (**a**) first cooling exotherm curves and (**b**) second heating exotherm curves of PPS/GF composites. (**c**) Calculated crystallinity of the PPS/GF composites from the DSC scan. (**d**) TGA thermographs of PPS/GF composites with different cooling temperatures.

The crystallization behavior of the PPS/GF composites resulting from their thermal properties was investigated via DSC analysis and compared with the XRD results (Figure 3 and Table 2). The melting (T_m_) and crystallization temperatures (T_c_) were measured by increasing the temperature for two cycles, and the degree of crystallinity (%X_c_) was calculated from the exothermic heat–flow curve of the DSC results to conduct a comparative analysis of the relative crystallinity. As shown in Figure 3, the melting points (T_m_) and crystallization temperatures (T_c_) of the PPS composites at each cooling temperature exhibited similar trends and gradually increased. The higher the cooling temperature, the finer were the densely stacked crystals; moreover, the melting enthalpy increases, resulting in an increase in crystallinity [11,28,29,30]. This is consistent with the XRD analysis of the crystallization behavior of the PPS/GF composites at different cooling temperatures. Accordingly, 160 °C was concluded to be the appropriate temperature for the injection mold temperature for the PPS composites, including 55 wt.% of GF with high crystallinity.

The thermal degradation behavior of the PPS/GF composites was investigated by TGA (Figure 3d and Table 3). The 5% weight loss (T_D_^95%^) temperature of the PPS/GF composites for each cooling temperature slightly increases as the cooling temperature increases, indicating high thermal stability owing to high chain stacking density and crystallinity. Furthermore, the temperature at which the first decomposition occurs (T_D1_) is between 417 and 426 °C, and the temperature at which PPS, the matrix, is decomposed (T_D2_) was confirmed to be approximately 578 °C on average. Conclusively, at a cooling temperature of 160 °C, the thermal decomposition temperature is the highest overall; thermal stability is also high. Moreover, over all conditions ramping the temperature to 900 °C, approximately 13% of inorganic materials, except for 55 wt.% of GF, remained.

### 3.3. Mechanical Properties

**Figure 4:**
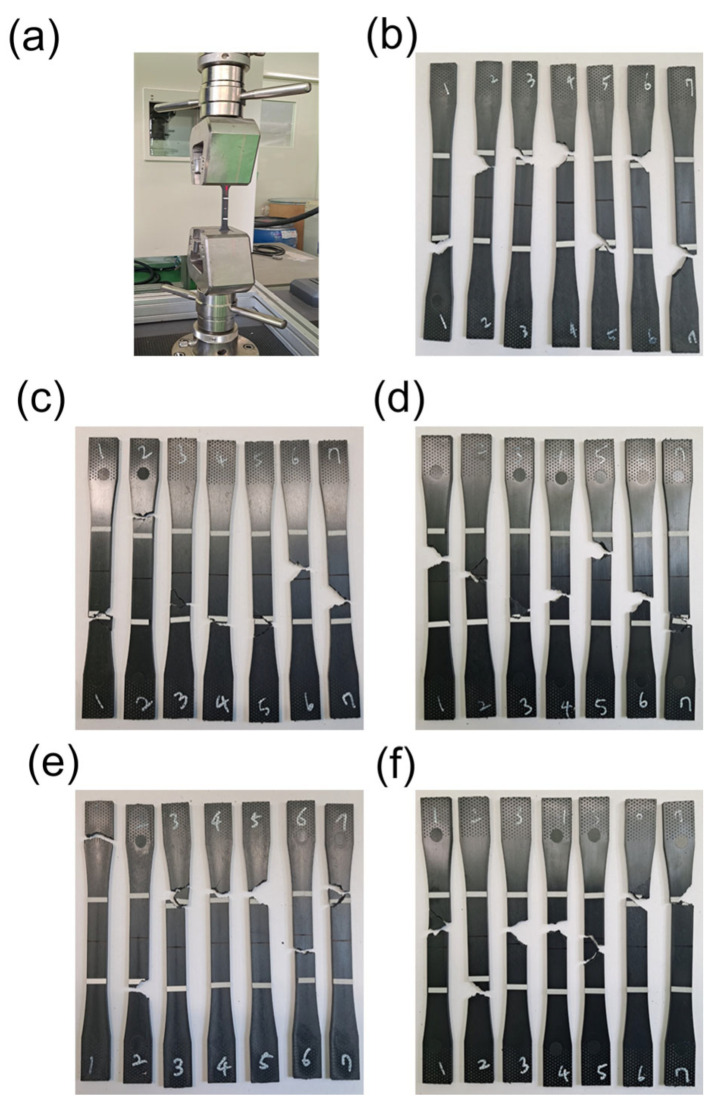
Photographs of (**a**) tensile test set up for PPS/GF composites and fractured specimen with different cooling temperatures of (**b**) 80 °C, (**c**) 100 °C, (**d**) 120 °C, (**e**) 140 °C, and (**f**) 160 °C, respectively.

**Figure 5:**
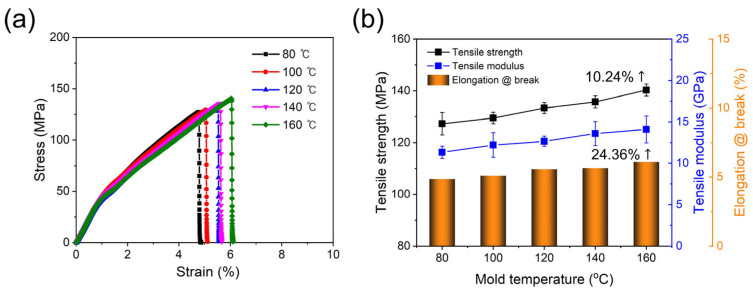
(**a**) Tensile stress–strain curves and (**b**) tensile strength, modulus, and elongation at break of PPS/GF composites with different cooling temperatures.

The tensile properties of the PPS/GF composites, which were injection-molded at different cooling temperatures, were investigated via tensile tests in accordance with ASTM D638 standards using a 100 kN load cell. Seven tests were performed for each case. As shown in Figure 4, the 55 wt.% composites show no visible differences in the fractured shape after the tensile test as the injection cooling temperature increases. However, as shown in Figure 5 and Table 4, as the cooling temperature increases, the tensile strength increases by approximately 10.24%: from 127 MPa at a cooling temperature of 80 °C to 140 MPa at 160 °C. Similarly, the tensile elastic modulus increases by 24.36%: from 11 GPa at a cooling temperature of 80 °C to 14 GPa at 160 °C. Thus, the PPS, a super engineering plastic with a higher processing temperature of 300 °C, exhibits excellent tensile properties at a cooling temperature of 160 °C. The elongation at break also shows a slight increase with an increase in cooling temperature.

**Figure 6:**
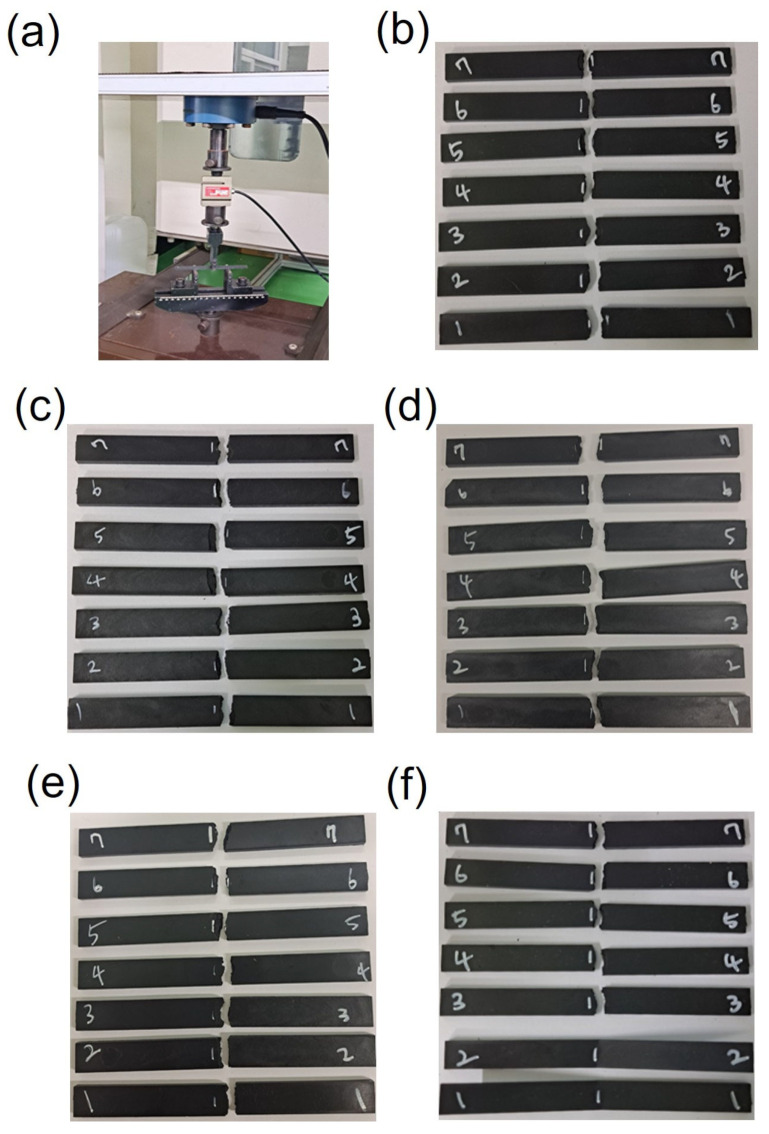
Photographs of (**a**) 3-point test set up for PPS/GF composites and fractured specimen with different cooling temperatures of (**b**) 80 °C, (**c**) 100 °C, (**d**) 120 °C, (**e**) 140 °C, and (**f**) 160 °C, respectively.

**Figure 7:**
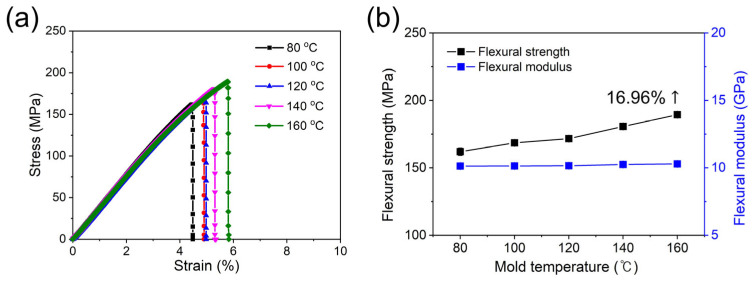
(**a**) Flexural stress–strain curves and (**b**) flexural strength, modulus of PPS/GF composites with different cooling temperatures.

The flexural strengths of the composites were determined using a 3-point bending test according to the ASTM D790 standard. As shown in Figure 6, the fractured sample with a cooling temperature of 160 °C shows that it withstands the bending load without fracture. As the injection cooling temperature increases for the PPS/GF 55 wt.% composites, the flexural strength initially exhibited a value of 162 MPa and increased gradually (Figure 7 and Table 5). However, cooling temperatures of 80 °C and 100 °C can cause defects in the composites due to the instability of the injection process for PPS, which has a glass transition temperature (T_g_) of approximately 120 °C. As a result, there is a low reproducibility of mechanical properties in the composites injected with a cooling temperature lower than 120 °C. The flexural behavior is consistent with the results of the tensile strength, showing an increasing trend with an increase in cooling temperature (162 MPa for the 80 °C case and 189 MPa for the 160 °C case, which is a 16.96% increase). However, the flexural moduli exhibited similar values for all conditions.

**Figure 8:**
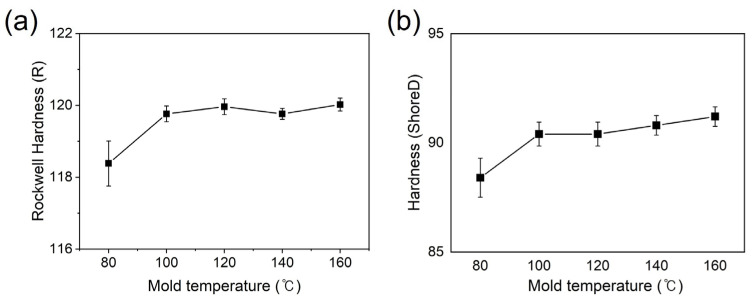
Hardness of PPS/GF composites with different cooling temperatures via (**a**) Rockwell method and (**b**) Shore method.

The hardness of the composites was investigated using the Rockwell and Shore hardness instruments. The Rockwell method was used to measure the hardness of the specimens by applying a constant load using a specified indenter and measuring the depth or area of the indentation. It is commonly used in plastic material testing with an R scale. Under the initial cooling temperature condition of 80 °C, the hardness value was approximately 118; while at a cooling temperature of 160 °C (twice the initial temperature), it slightly increased to 120 (Figure 8a). As the cooling temperature increases, the PPS crystalline structure is expected to become finer and harder, resulting in increased hardness. The Shore-D hardness testing method was used to evaluate the surface hardness of the plastics, with higher values indicating harder plastics. Similar to the Rockwell results, the hardness of the PPS/GF composites increased with increasing injection cooling temperature, as shown in Figure 8b. At the initial cooling temperature condition of 80 °C, a hardness value of 88.4 was obtained, while at 160 °C, it showed an increasing trend, reaching 91.2. This indicated an increase in the surface hardness as the polymer crystallinity increased.

**Figure 9:**
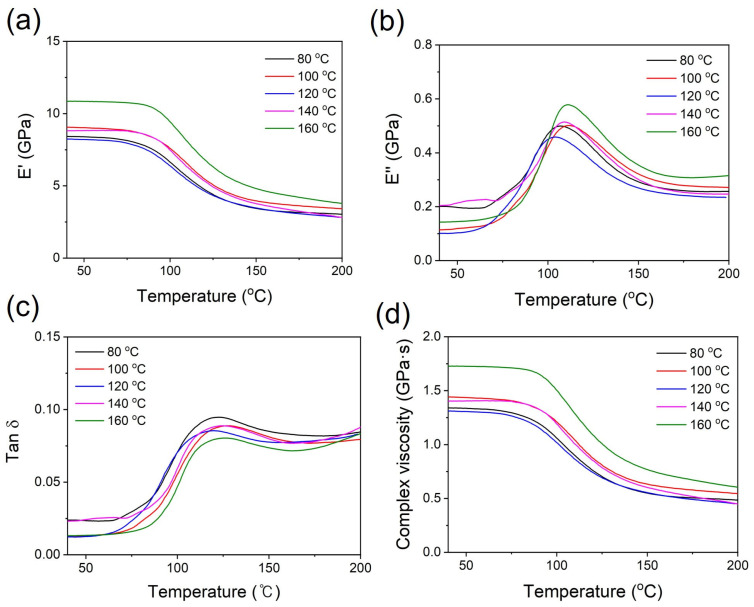
(**a**) Storage modulus (E′), (**b**) loss modulus (E″), (**c**) tan δ and (**d**) complex viscosity of the PPS/GF composites with different cooling temperatures.

Dynamic mechanical analysis (DMA) of the PPS/GF composites was conducted to analyze their elastic stiffness as a function of temperature. This method enables the investigation of the changes in the elastic and viscous properties of the PPS/GF composites during annealing, providing a qualitative comparison of the crystallization behavior resulting from polymer chain mobility [31]. As shown in Figure 9, the PPS/GF composites exhibit an increase in the storage modulus E′ (elasticity) with increasing cooling temperature, leading to a decrease in tan delta. This trend is particularly significant at the 160 °C condition showing E′ of 10.85 GPa, representing a 30% improvement compared to the 80 °C case (8.36 GPa). Additionally, the glass transition temperature (T_g_) measured at the peak of the tan delta curve showed a gradual increase with increasing cooling temperature (Figure 9c and Table 6). Similar to the DSC results, it can be inferred that as the cooling temperature increased, the fine crystalline structure restricted the mobility of the polymer chains, resulting in increased matrix rigidity. Therefore, the optimal injection cooling (mold) temperature for the mechanical properties of the PPS/GF 55 wt.% composites is 160 °C.

**Figure 10:**
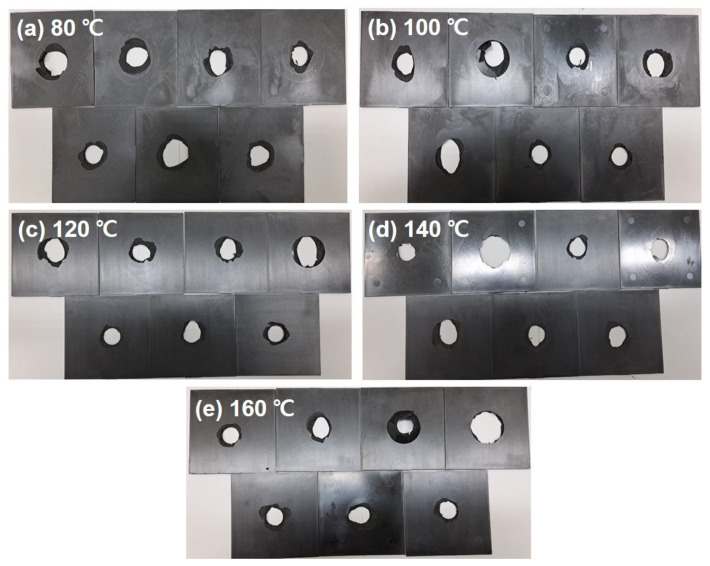
Photographs fractured PPS/GF composite specimen through drop-weight impact test with different cooling temperatures of (**a**) 80 °C, (**b**) 100 °C, (**c**) 120 °C, (**d**) 140 °C and (**e**) 160 °C, respectively.

**Figure 11:**
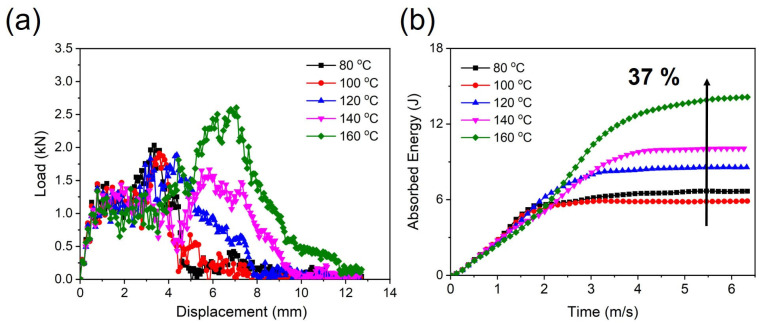
Impact behavior of the PPS/GF composites: (**a**) load vs. displacement curve resulting in (**b**) 37% increase in energy absorption compared with the control sample.

Furthermore, Figure 10 shows photographs of the fractured PPS/GF composites after the drop-weight impact test. The impact test results show that the composites exhibited severe penetration in all test cases. The absorbed energy behaviors of the composites were analyzed using a drop-weight impact test, as shown in Figure 11. The combination of the elastic portion caused by a high GF load, the interface between the PPS matrix and GFs, and the matrix itself being prone to fracture contributed to the observed fluctuation curves in all cases (Figure 11a). In particular, the sample under the 160 °C cooling temperature exhibits a characteristic behavior of a material with higher elasticity than the other samples. Thus, as the cooling temperature increases, the absorbed energy also increases. There is an approximately 37% increase in absorbed energy from the 80 °C case to the 160 °C case. Hence, it is necessary to select a higher cooling temperature for stable injection molding of PPS, including 55 wt.%, as it has been observed that as the injection cooling temperature increases up to 160 °C, there is an overall improvement in energy absorption behavior without any injection error.

### 3.4. Fractured Morphology Analysis of PPS/GF Composites

**Figure 12:**
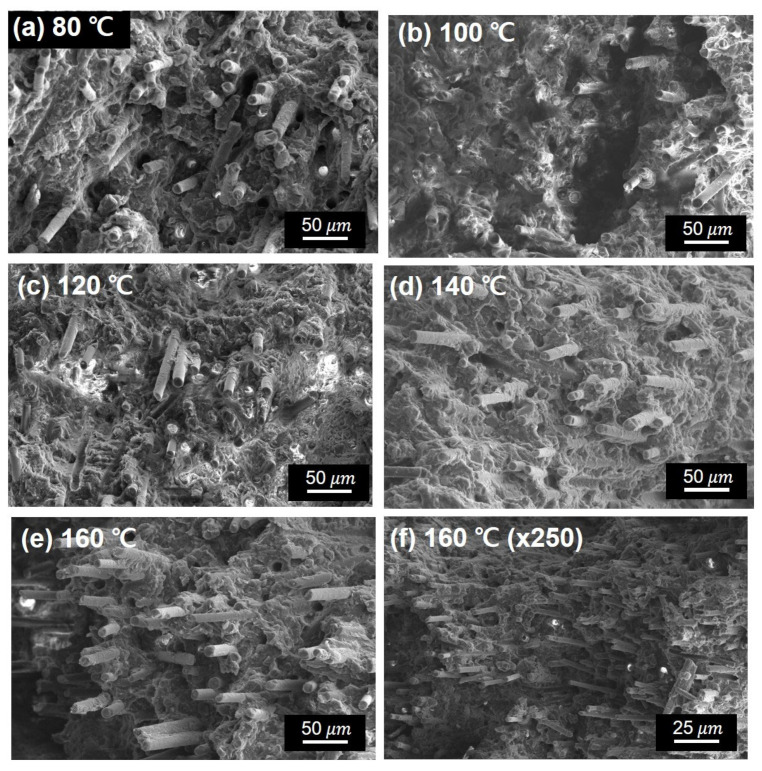
Cross-section SEM images of fractured PPS/GF composites with different cooling temperatures of (**a**) 80 °C, (**b**) 100 °C, (**c**) 120 °C, (**d**) 140 °C, (**e**) 160 °C, and (**f**) ×250 low magnified of (**e**).

The FE-SEM images (Figure 12) show the cross-sectional morphologies of the fractured PPS/GF composites. SEM analysis of the fracture surface after drop weight impact testing revealed that under the low injection cooling temperature of 80 °C, the PPS composite could have a relatively short cooling time. This led to a loosely formed crystalline structure because the PPS resin had limited time to cool and crystallize. Additionally, a lower orientation of the GF was observed, and the resin impregnation state was relatively poor, resulting in fiber pull-out behavior. In other words, as the injection cooling temperature increased, the orientation of the chopped GF also increased. This resulted in improved resin impregnation and relatively slower crystallization of PPS. The increased cooling temperature provides sufficient time for the polymer chains to crystallize, which can contribute to the enhancement of the mechanical properties of the composites, particularly by strengthening the interphase between the fiber and resin.

## 4. Conclusions

In this study, the correlation between the crystallinity behavior of PPS in the composites and the mechanical properties of the composites according to the cooling temperature for injection molding of the PPS/GF 55 wt.% composites was investigated. Increasing the injection cooling temperature enhanced the degree of crystallization in the PPS matrix. This phenomenon is attributed to the crystallization of the polymer chains, which can improve the mechanical properties of the composite. Furthermore, the alignment of the chopped GF also increased, leading to enhanced interfacial reinforcement between the fibers and the matrix, thereby improving the overall quality of the molded parts. Lower cooling temperatures result in shorter cooling times for the resin, leading to loosely formed crystalline structures. This was observed to negatively impact the resin impregnation and contribute to the fiber pull-out behavior. In conclusion, by controlling the appropriate cooling temperature for the injection molding process, it is possible to promote interfacial reinforcement between the fibers and the matrix and facilitate resin crystallization, thereby achieving superior mechanical performance. These findings provide insights into the correlation between the cooling temperature during injection molding and the mechanical properties of the PPS/GF 55 wt.% composite, aiding in the optimization of the injection molding process.

## Figures and Tables

**Table 1:** Crystallization properties of the (110, 200) reflections in PPS/GF composites with different cooling temperatures based on XRD analysis.

Cooling Temperature (°C)	2θ (110, 200)	FWHM (110, 200)	Crystallite Size (nm)	CI ^a^ (%)
80	19.1	6.38	1.32	18.76
100	20.1	3.17	2.66	22.88
120	20.4	1.41	2.87	25.46
140	20.3	1.54	3.96	27.54
160	20.3	2.12	5.98	36.94

**^a^** Calculated using Equation (2).

**Table 2:** DSC data and crystallinities of PPS/GF composites with different cooling temperatures.

Cooling Temperature (°C)	T_m_ (°C)	ΔH_m_^b^ (J/g)	T_c_ (°C)	ΔH_c_^b^ (J/g)	Χ_c_^c^ (%)
80	281.67	16.65	233.51	18.67	24.6
100	281.83	17.86	233.88	20.10	26.4
120	281.67	15.92	233.01	17.57	23.4
140	283.50	16.76	234.52	18.94	24.8
160	282.51	19.34	234.19	20.39	28.6

**^b^** Enthalpies of the PPS/GF composites from DSC analysis software. **^c^** Calculated using Equation (3).

**Table 3:** 5 wt.% loss and degradation temperature of PPS/GF composites with different cooling temperatures.

Cooling Temperature (°C)	5% Weight Loss Temperature, T_D_^5%^ (℃)	T_D1_ (°C)	T_D2_ (°C)
80	500.72	417.75	574.31
100	505.32	420.56	577.84
120	503.41	421.79	577.84
140	509.08	421.84	583.70
160	512.87	426.95	583.97

**Table 4:** Tensile strength and modulus of PPS/GF composites with different cooling temperatures.

Cooling Temperature (°C)	Tensile Strength (MPa)	Tensile Modulus (GPa)	Elongation @ Break (%)
80	127.29 ± 4.34	11.33 ± 0.74	4.87
100	129.51 ± 2.23	12.21 ± 1.47	5.11
120	133.35 ± 2.14	12.66 ± 0.60	5.58
140	135.73 ± 2.34	13.59 ± 1.45	5.66
160	140.32 ± 2.30	14.09 ± 1.62	6.09

**Table 5:** Flexural strength and modulus of PPS/GF composites with different cooling temperatures.

Cooling Temperature (°C)	Flexural Strength (MPa)	Flexural Modulus (GPa)
80	161.95 ± 2.77	10.13 ± 0.19
100	168.52 ± 0.12	10.14 ± 0.13
120	171.68 ± 1.38	10.15 ± 0.03
140	180.63 ± 1.37	10.25 ± 0.06
160	189.42 ± 1.54	10.29 ± 0.18

**Table 6:** Storage modulus (E′) and tan δ analysis of PPS/GF composites with different cooling temperatures.

Cooling Temperature (°C)	E′ at 40 °C (GPa)	T_g_ (°C)	tanδ _max_
80	8.36	122.81	0.094
100	9.05	127.73	0.088
120	8.25	119.76	0.085
140	8.81	124.48	0.088
160	10.85	128.25	0.080

## Data Availability

All data used during the study appear in the submitted article.
